# [^18^F] Sodium Fluoride Dose Reduction Enabled by Digital Photon Counting PET/CT for Evaluation of Osteoblastic Activity

**DOI:** 10.3389/fmed.2021.725118

**Published:** 2022-01-12

**Authors:** Maria I. Menendez, Richard R. Moore, Mahmoud Abdel-Rasoul, Chadwick L. Wright, Soledad Fernandez, Rebecca D. Jackson, Michael V. Knopp

**Affiliations:** ^1^Department of Radiology, The Wright Center of Innovation in Biomedical Imaging, The Ohio State University Wexner Medical Center, Columbus, OH, United States; ^2^Center for Biostatistics, College of Medicine, The Ohio State University, Columbus, OH, United States; ^3^Department of Internal Medicine, Endocrinology, Diabetes and Metabolism, The Ohio State University Wexner Medical Center, Columbus, OH, United States

**Keywords:** [^18^F] sodium fluoride, PET/CT, digital photon counting, bone imaging, bone metabolism, sodium fluoride dose reduction, preclinical molecular imaging, canine PET

## Abstract

The aim of the study was to assess the quality and reproducibility of reducing the injected [^18^F] sodium fluoride ([^18^F]NaF) dose while maintaining diagnostic imaging quality in bone imaging in a preclinical skeletal model using digital photon counting PET (dPET) detector technology. Beagles (*n* = 9) were administered three different [^18^F]NaF doses: 111 MBq (*n* = 5), 20 MBq (*n* = 5), and 1.9 MBq (*n* = 9). Imaging started ≃45 min post-injection for ≃30 min total acquisition time. Images were reconstructed using Time-of-Flight, ultra-high definition (voxel size of 1 × 1 × 1 mm^3^), with 3 iterations and 3 subsets. Point spread function was modeled and Gaussian filtering was applied. Skeleton qualitative and quantitative molecular image assessment was performed. The overall diagnostic quality of all images scored excellent (61%) and acceptable (39%) by all the reviewers. [^18^F]NaF SUV_mean_ showed no statistically significant differences among the three doses in any of the region of interest assessed. This study demonstrated that a 60-fold [^18^F]NaF dose reduction was not significantly different from the highest dose, and it had not significant effect on overall image quality and quantitative accuracy. In the future, ultra-low dose [^18^F]NaF dPET/CT imaging may significantly decrease PET radiation exposure to preclinical subjects and personnel.

## Introduction

Sodium Fluoride ([^18^F]NaF) Positron Emission Tomography—Computed Tomography (PET/CT) is used clinically in oncology patients to detect and characterize osteoblastic metastatic lesions (1–4), as well as to aid visualization of atherosclerotic calcifications and plaques in patients with cardiovascular disease (5–7). Recently, [^18^F]NaF has also been used as a bone imaging biomarker to assess and quantify bone metabolic processes (i.e., osteoblastic activity) in non-oncologic musculoskeletal disorders such as osteoarthritis and osteoporosis (8–20). The radiolabeled fluoride ion exchanges with the hydroxyl groups in hydroxyapatite crystals on the surface of the bone matrix to form fluoroapatite. Therefore, uptake of [^18^F]NaF uptake can be used a marker of osteoblastic bone metabolism. [^18^F]NaF PET/CT imaging is a sensitive, noninvasive, imaging approach to assess bone metabolism (21–23). Due to its [^18^F]NaF favorable pharmacokinetics, such as high bone uptake, minimal binding to serum proteins, rapid single-pass extraction, and fast clearance from the soft tissues, [^18^F]NaF PET is more sensitive for detecting abnormal osteoblastic activity and lesions than the current clinical gold standard, ^99m^Technetium-labeled methylene diphosphonate (^99m^Tc-MDP) gamma scintigraphy. When compared with ^99m^Tc-MDP gamma scintigraphy, [^18^F]NaF PET has higher sensitivity, superior image resolution, and improved target-to-background ratio (24). Hybrid imaging modalities such as PET/CT and PET/Magnetic Resonance Imaging (MRI) are currently used for assessing bone metabolism (22–25). PET/MRI presents operational challenges including attenuation correction for PET, longer than desired MRI image acquisition times when compared with CT, and reduced field of view when using dedicated MRI coils (e.g., knee coils) which fail to aid in whole body skeletal assessment (25–27). Radiation exposure can be a concern due to ionizing radiation from PET radiotracers (28). Minimizing radiation exposure is important not only for research subjects and patients, but also for imaging technologists, nursing personnel and subject/patient caregivers. It is believed that significant reductions in PET radiotracer doses will benefit pediatric subjects/patients and those subjects/patients participating in longitudinal studies with multiple serial PET studies by reducing cumulative radiation exposure (29, 30). Many imaging studies have focused on CT radiation dose reduction without addressing the potential dose reduction strategies associated with the administered PET radiotracers (31, 32). Most PET radiotracer dose reduction has been focused on the widely clinically utilized radiotracer: 2-deoxy-2-[^18^F]-fluorodeoxyglucose (^18^F-FDG) (33–36). The current recommended guidelines for human [^18^F]NaF injected doses from the European Association of Nuclear Medicine (EANM) are weight based. For Adults: 1.5–3.7 MBq /kg (megabecquerel (MBq) per kilogram (kg) of body weight (BW), and Pediatrics: 2.2 MBq/kg (37). The Society of Nuclear Medicine and Molecular Imaging (SNMMI) guidelines recommend a fixed dose for adults: 185–370 MBq, and weight-based for Pediatrics (2.22 MBq/kg) (38). Some clinical studies have examined [^18^F]NaF dose reduction and reported no effect on image quality (30, 39–41). However, comprehensive preclinical [^18^F]NaF dose reduction studies in translational large animal models and its impact on overall PET image quality are missing.

The recent introduction of clinically approved PET/CT systems equipped with digital photon counting PET (dPET) detector technology enables new PET imaging approaches for addressing PET radiotracer dose reduction, faster PET image acquisition times, and higher definition in PET image reconstruction (42, 43). Digital photon counting PET detector technology enables ultra-high definition reconstruction with voxel volume of 1 × 1 × 1 mm^3^ and more precisely localizes PET annihilation events (i.e., reduces partial volume effects) which improve quantitative PET accuracy for imaging biomarker assessment (44–46). Additionally, lower PET doses can be implemented in dPET imaging in accordance with ALARA (As Low as Reasonably Achievable) while maintaining diagnostic imaging quality (47–49). With the recent advances in dPET detector technology, there is an immediate opportunity to minimize PET radiotracer doses in preclinical research subjects imaged on these clinical dPET/CT systems and likewise reduce radiation exposures to PET staff and handling personnel. This study is an important step to develop and standardize low-dose hybrid PET-CT imaging methodologies in preclinical imaging, and to provide guidance for future clinical studies, and clinical trials applying [^18^F]NaF PET dose reduction.

The aim of the study was to assess the quality and reproducibility of reducing the injected [^18^F]NaF dose while maintaining diagnostic imaging quality in bone imaging in a preclinical skeletal model using digital photon counting PET (dPET) detector technology.

We hypothesized that 5- to 60-fold reductions in administered [^18^F]NaF activity would provide equivalent image quality on dPET/CT when compared with the standard [^18^F]NaF doses.

## Materials and Methods

### Animals

This study was conducted according to NIH guidelines, and according to protocols approved by the Institutional Animal Care and Use Committee (IACUC) of The Ohio State University. Nine healthy skeletally mature male beagles [weight (kg) mean ± SEM; 15 ± 4.7] were used.

### Positron Emission Tomography/Computed Tomography Acquisition

Subjects underwent general anesthesia induced by acepromazine [Aceproject; Henry Schein Animal Health, Dublin OH; intravenously (IV), 0.1 mg/kg], ketamine (Ketasthesia; Henry Schein Animal Health, Dublin OH; IV, 10 mg/kg), and diazepam (Hospira; Lake Forest, IL; IV, 0.25 mg/kg) and maintained by isoflurane (Isothesia; Henry Schein Animal Health, Dublin, OH; 1–4%). The subjects were place in supine position with the front and distal extremities extended and supported in a custom-made multimodal imaging positioning device to mimic human scans and to improve the precision and positional consistency among scans (13, 50). Subjects were intravenously administered 3 different [^18^F]NaF target doses: 111 MBq (standard dose/SD; *n* = 5), 20 ± 7.8 MBq [mean ± standard error of the mean (SEM); low dose/LD; *n* = 5], and 1.9 MBq (ultra- low dose/ULD; *n* = 9) (see Table 1).

**Table 1:** Dogs and corresponding standard (SD), low (LD) [mean ± standard error of the mean (SEM)], and ultra-Low (ULD) [^18^F]NaF doses.

**Dog**	**Standard** **dose** **(111 MBq)**	**Low dose (20** **±** **7.8 MBq)**			**Ultra-low** **dose** **(1.9 MBq)**
		**37 MBq**	**18.5 MBq**	**3.7 MBq**	
111		X			X
112			X		X
113		X		X	X
114	X			X	X
115					X
116	X				X
117	X				X
118	X				XX
107	X				

**Figure 1:**
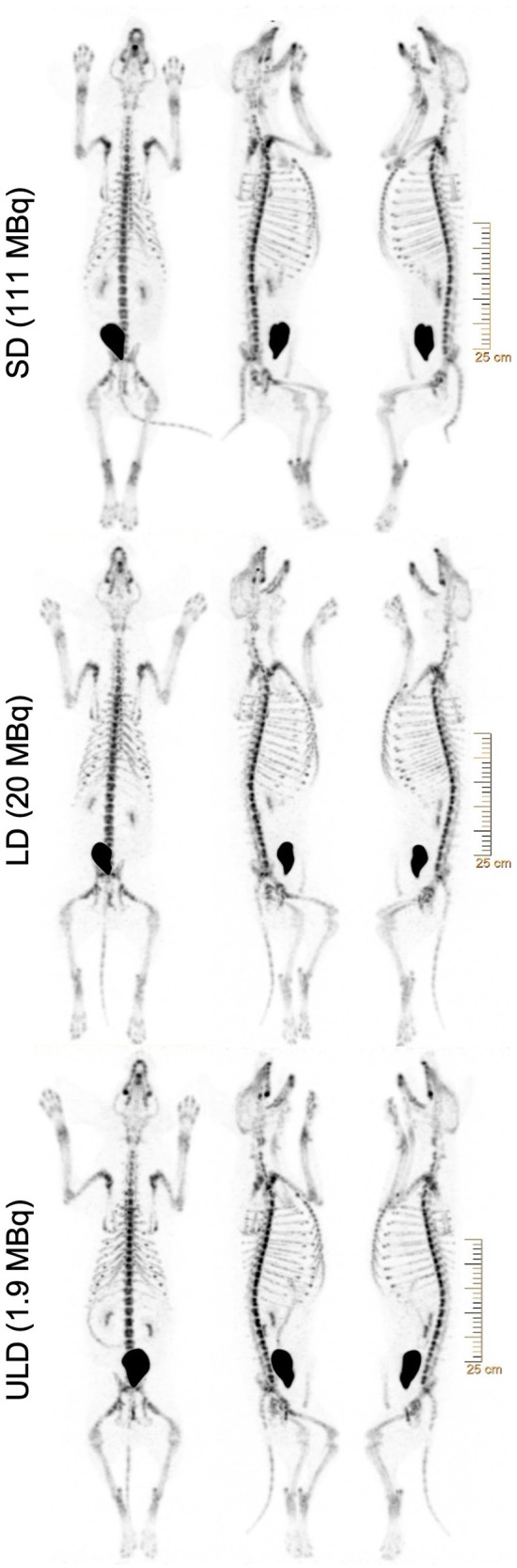
Representative whole-body [^18^F]NaF PET maximum intensity projections (MIPs) REDCap surveys showing the same subject with standard (SD), low (LD), and ultra-low dose (ULD) from top to bottom. Subject scans were presented with three maximum intensity projections (MIPs), with 0°, 108°, and −90° angles (from left to right), with a gray level of 10.00. All images were reconstructed using ultra-high-definition (voxel volume = 1 × 1 × 1 mm^3^), 3 iterations, and 3 subsets. Point spread function (PSF) was modeled and Gaussian filtering was applied. SD and LD were retrospectively list-mode clipped accordingly to simulate the same count density as ULD.

**Figure 2:**
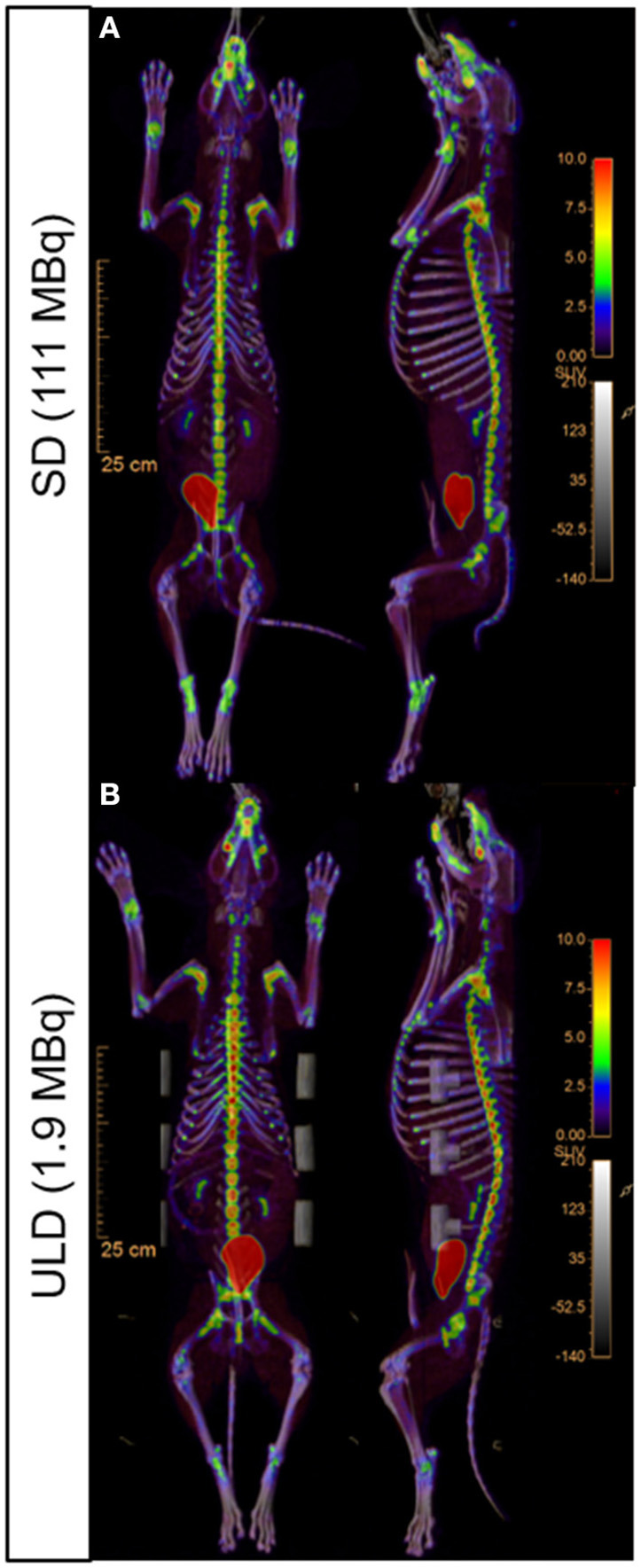
Representative whole-body [^18^F]NaF PET/CT uptake fusion showing the metabolic bone activity. Standard dose [SD; **(A)**] and ultra-low dose [ULD; **(B)**] dorsal (left) and sagittal (right) images.

All imaging was performed using the Vereos dPET/CT system (Philips, Cleveland, Ohio). A low-dose computed tomography (CT) scan was performed for attenuation correction and coregistration. For the low dose (LD) and ultra-low dose (ULD), whole-body PET imaging began at ~45 min post-injection using acquisitions times of 180 s/bed position in list-mode for 10 bed positions (total dPET image acquisition time ~30 min). The standard dose (SD) (*n* = 5) began at ~30 min and was acquired with 120 sec/bed. All SD dPET acquisitions were retrospectively list-mode clipped from 120 s/bed to 4 s/bed to simulate the same count density as ULD. In addition, all LD dPET acquisitions were retrospectively list-mode clipped from 180 s/bed to 18 s/bed to simulate the same count density as ULD. All list-mode clipped SD, list-mode clipped LD, and ULD acquisitions were reconstructed using Time-of-Flight and ultra-high-definition (voxel volume = 1 × 1 × 1 mm^3^), three iterations, and three subsets. Point spread function (PSF) was modeled and Gaussian filtering was applied (Figures 1, 2) (Supplementary Material).

### Qualitative Image Analysis

Philips Intellispace Portal was used to generate the images for subsequent review by a blinded reader panel using REDCap survey platform. Each dPET scan was presented with three non-rotating maximum intensity projection (MIP) images, with 0° (ventral projection), 108° (right posterior oblique projection) and−90° (left lateral projection) angles, with a PET SUV window level of 0–10. REDCap surveys recorded the reader assessment of [^18^F]NaF dPET image quality (Figure 1). The images were reviewed by three authors (CW, MVK, MIM) working independently and blinded to radiotracer dose administered. Reviewers performed imaging assessment of the entire imaging dataset twice with at least 1 week between reader assessments to control for visual memory. The image datasets were randomly ordered between surveys.

Qualitative reader assessment outcome parameters included: overall diagnostic quality of the images, and regional bone [^18^F]NaF uptake of the following regions of interest (ROI): cervical, thoracic, and lumbar, spine, skull, proximal long bones (scapula, humerus, and radius and ulna), distal long bones (femur, tibia, and pelvis), short bones (carpal joint bones, metacarpal bones, tarsal joint bones, metatarsal bones, proximal, middle, and distal phalanges) (Table 2).

**Table 2:** Qualitative image analysis as score frequencies and percentages (%) for the overall image quality and the skeletal regions of interest using standard (SD), low (LD), and ultra-low (ULD) [^18^F]NaF doses.

**Region of interest**	**Score**	**Standard** **dose** **(111 MBq)**	**Low** **dose** **(20 MBq)**	**Ultra-low** **dose** **(1.9 MBq)**
**Overall image quality**				
	Excellent	25 (62.5%)	24 (60.0%)	39 (54.2%)
	Acceptable	15 (37.5%)	16 (40.0%)	33 (45.8%)
	Insufficient	0 (0.0%)	0 (0.0%)	0 (0.0%)
	Not acceptable	0 (0.0%)	0 (0.0%)	0 (0.0%)
**Cervical spine**				
	Excellent	16 (40.0%)	20 (50.0%)	34 (47.2%)
	Acceptable	22 (55.0%)	18 (45.0%)	38 (52.8%)
	Insufficient	2 (5.0%)	2 (5.0%)	0 (0.0%)
	Not acceptable	0 (0.0%)	0 (0.0%)	0 (0.0%)
**Thoracic spine**				
	Excellent	40 (100.0%)	39 (97.5%)	69 (97.2%)
	Acceptable	0 (0.0%)	1 (2.5%)	2 (2.8%)
	Insufficient	0 (0.0%)	0 (0.0%)	0 (0.0%)
	Not acceptable	0 (0.0%)	0 (0.0%)	0 (0.0%)
**Lumbar spine**				
	Excellent	40 (100.0%)	39 (97.5%)	70 (97.2%)
	Acceptable	0 (0.0%)	1 (2.5%)	2 (2.8%)
	Insufficient	0 (0.0%)	0 (0.0%)	0 (0.0%)
	Not acceptable	0 (0.0%)	0 (0.0%)	0 (0.0%)
**Skull**				
	Excellent	15 (37.5%)	15 (38.5%)	27 (37.5%)
	Acceptable	25 (62.5%)	24 (61.5%)	45 (62.5%)
	Insufficient	0 (0.0%)	0 (0.0%)	0 (0.0%)
	Not acceptable	0 (0.0%)	0 (0.0%)	0 (0.0%)
**Proximal long bones**				
	Excellent	18 (45.0%)	17 (42.5%)	28 (38.9%)
	Acceptable	22 (55.0%)	23 (57.5%)	44 (61.1%)
	Insufficient	0 (0.0%)	0 (0.0%)	0 (0.0%)
	Not acceptable	0 (0.0%)	0 (0.0%)	0 (0.0%)
**Distal long bones**				
	Excellent	19 (47.5%)	19 (48.7%)	27 (37.5%)
	Acceptable	21 (52.5%)	20 (51.3%)	42 (58.3%)
	Insufficient	0 (0.0%)	0 (0.0%)	3 (4.2%)
	Not acceptable	0 (0.0%)	0 (0.0%)	0 (0.0%)
**Short bones**				
	Excellent	20 (50.0%)	20 (50.0%)	32 (44.4%)
	Acceptable	20 (50.0%)	16 (40.0%)	40 (55.6%)
	Insufficient	0 (0.0%)	4 (10.0%)	0 (0.0%)
	Not acceptable	0 (0.0%)	0 (0.0%)	0 (0.0%)

*Reviewer's scores are combined*.

The qualitative [^18^F]NaF uptake was scored 1–4 (1 = not acceptable; 2 = insufficient; 3 = acceptable; 4 = excellent).

### Semi-Quantitative Image Analysis

Maximum and mean standardized uptake values (SUV_max_ and SUV_mean_) for [^18^F]NaF activity were assessed using two-dimensional (2D) ROI, manually traced over representative osseous structures including mandible, carpus, first lumbar vertebral body, distal femur, tarsus, caudal vertebrae, and a region of the liver (Tables 3, 4).

**Table 3:** [^18^F]NaF mean standardized uptake values (SUV_mean_) of regions of interest (ROIs).

	**Standard dose** **(111 MBq)**	**Low** **dose** **(20 MBq)**	**Ultra-low** **dose** **(1.9 MBq)**	**Overall** ***p*****-value**
ROI	Mean (95%CI)	Mean (95%CI)	Mean (95%CI)	
First lumbar	8.6 (7.0, 10.1)	8.8 (7.3, 10.4)	9.2 (8.0, 10.3)	0.7723
Distal femur	3.1 (2.6, 3.6)	3.1 (2.6, 3.6)	2.5 (2.1, 2.9)	0.0528
Carpus	3.2 (2.2, 4.1)	2.8 (1.8, 3.9)	2.9 (2.2, 3.6)	0.8394
Mandible	2.5 (2.1, 3.0)	2.3 (1.9, 2.8)	2.5 (2.1, 2.9)	0.6241
Tarsus	2.9 (2.2, 3.6)	2.6 (1.9, 3.3)	2.1 (1.6, 2.7)	0.2124
Caudal vertebrae	0.7 (0.2, 1.2)	1.2 (0.7, 1.7)	1.2 (0.9, 1.6)	0.1776
Liver	0.6 (0.4, 0.9)	0.5 (0.2, 0.8)	0.7 (0.5, 0.9)	0.4803

[^18^F]NaF, sodium fluoride; CI, confidence intervals. ^*^p < 0.05 considered significant.

**Table 4:** [^18^F]NaF maximum standardized uptake values (SUV_max_) of regions of interest (ROIs).

	**Standard** **dose** **(111 MBq)**	**Low** **dose** **(20 MBq)**	**Ultra-low** **dose** **(1.9 MBq)**	**Overall** ***p*****-value**
ROI	Mean (95%CI)	Mean (95%CI)	Mean (95%CI)	
First lumbar	14.8 (11.7, 17.9)*	19.6 (16.5, 22.7)	19.7 (17.3, 22.0)	0.0380
Distal femur	4.6 (3.8, 5.3)	4.1 (3.1, 5.0)	3.6 (3.0, 4.3)	0.1025
Carpus	7.2 (5.4, 9.1)	7.7 (5.7, 9.8)	7.2 (5.8, 8.6)	0.8861
Mandible	6.9 (5.6, 8.2)	7.4 (5.8, 8.9)	8.6 (7.6, 9.6)	0.0821
Tarsus	6.3 (4.9, 7.7)	7.4 (6.0, 8.8)	6.1 (5.1, 7.2)	0.3149
Caudal vertebrae	6.3 (4.2, 8.5)	6.2 (3.5, 8.9)	6.2 (4.6, 7.9)	0.9951
Liver max	0.7 (0.4, 1.0)	0.6 (0.3, 1.0)	1.0 (0.8, 1.2)	0.1428

[^18^F]NaF, sodium fluoride; CI, confidence intervals. ^*^p < 0.05 considered significant.

### Statistical Analysis

Reviewer ratings of images are reported as rating frequencies and percentages for each ROI are represented in Table 2. Continuous outcome variables for dPET: SUV_max_, and SUV_mean_ were modeled using linear mixed models with random intercepts and categorical fixed effects representing dose (Tables 3, 4). The random intercepts account for correlation between repeated measures on each canine. Results are reported as model based estimated means and 95% confidence intervals. Overall *p*-values for groups effects are also reported. All hypothesis tests were conducted at a 5% type I error level. All statistical analyses were conducted using SAS version 9.4 (SAS Institute, Cary, NC).

## Results

All dPET/CT imaging studies were completed and all dPET image datasets (*n* = 19) were deemed evaluable.

### Qualitative Image Analysis

The overall diagnostic quality of all images was scored as excellent (61%) and acceptable (39%) by the three reviewers (Table 2). The skull images scored excellent (66%) and acceptable (34%). Both thoracic and lumbar spine images were scored excellent (98%) and acceptable (2%). The proximal long bones images were scored excellent (61%) and acceptable (39%). Cervical spine images were scored excellent (43%), acceptable (54%), and insufficient (4%). Distal long bones images were scored excellent (41%), acceptable (58%), and insufficient (1%). Short bones images were scored excellent (46%), acceptable (52%), and insufficient (2%). Only between 1 and 4% of the cervical spine, distal long bones, and short bones images were scored as insufficient [^18^F]NaF uptake compared to 96–99% acceptable and excellent uptake scores. None of the ROI images were scored as not acceptable. Table 2 summarizes the reviewer's score frequency for each skeletal ROI.

### Semi-Quantitative Image Analysis

Sodium fluoride ([^18^F]NaF) SUV_mean_ showed no statistically significant differences among the three doses (SD, LD, and ULD) in any of the osseous structures assessed (i.e., mandible, carpus, first lumbar vertebra, distal femur, tarsus, caudal vertebrae, and a region of the liver) (Table 3). For the [^18^F]NaF SUV_max_ (Table 4), only the first lumbar vertebra showed statistically significant differences among the three doses with the SUV_max_ at SD significantly lower than the SUV_max_ values at LD (*p* < 0.03) and ULD (*p* < 0.02). The mandible, carpus, distal femur, tarsus, caudal vertebrae, and a region of the liver showed no statistically significant differences among the three doses.

## Discussion

This study demonstrates that a 60-fold sodium fluoride ([^18^F]NaF) dose (ULD) reduction did not significantly differ in image quality and quantification compared to the standard dose (SD) in a healthy canine model. Our findings present a feasible option to markedly reduce [^18^F]NaF radiotracer doses in a translational preclinical system of bone imaging using a dPET/CT system without loss of overall imaging quality.

Regardless of the [^18^F]NaF dose, the overall dPET image quality assessment demonstrated diagnostic image quality in all [^18^F]NaF dPET image data sets with 61% of the scans scored as excellent and 39% scored as acceptable. No dPET imaging study received an insufficient or not acceptable score. These results indicate that ULD [^18^F]NaF dPET image quality was comparable to SD and even LD [^18^F]NaF dPET images (Table 2). ULD imaging is readily achieved with the new dPET detector capabilities enabled by improved spatial and temporal resolutions, reduced dead time, and higher dynamic count rate range when compared with conventional, analog photomultiplier tube based PET (cPET) detectors which has been previously described (43, 45, 46, 51, 52). In existing cPET systems, multiple scintillation crystals are coupled to multiple photomultiplier tube-based detectors whereas each scintillation crystal is coupled 1:1 with a single digital photon counting dPET detector. Hence, the combination of the direct coupling (1:1), with the enhanced time of flight (TOF) improves the timing and volumetric resolutions of the digital over the analog PET (44–46). The advantages of the dPET compared to cPET, leveraged with the reconstruction capabilities that allow for a 64-fold matrix reduction (from 4 × 4 × 4 to 1 × 1 × 1 mm^3^) favor radiation exposure reduction without sacrificing image quality.

It allows us to move preclinical nuclear medicine imaging forward with substantial reduced exposure levels while preserving image quality.

This study showed that using a clinical dPET/CT system in a large animal model might provide guidance to perform translational studies that currently are only feasible in small laboratory animals using specialized micro PET/CT systems.

In the regional image quality assessment, the skull, thoracic and lumbar spine, and proximal long bones were scored excellent and acceptable, with the thoracic and lumbar spine scoring the highest on image quality. This finding is consistent with a study in skeletally immature healthy canines (53), and a human study of the spine with [^18^F]NaF in healthy individuals were thoracic and lumbar spine had significant higher uptake compared to the cervical spine (54). An intriguing finding of the current study was that the cervical spine images were scored excellent and acceptable for most of the samples (96%) and 4% were scored insufficient. The atlas (C1) and the axis (C2) showed an uptake similar to the skull and lower than the rest of the cervical vertebrae (C3–C7) (Table 2). This may be due to anatomical differences and blood perfusion in those vertebrae, and the fact that C1–C2 lack vertebral bodies and marrow cavities, which provide capacity for higher blood perfusion (and hence bone radiotracer uptake), in addition to have anatomically different spinous and transverse processes. The short bones images were scored excellent and acceptable (98%) with a 2% that were scored insufficient, this small percentage may be due to the lower uptake of the distal phalanges due to a relatively decreased peripheral blood flow which leads to less radiotracer availability regionally in these areas when compared with the axial skeletal structures. Additionally, if the distal extremities were relatively colder to the axial skeleton, peripheral vasoconstriction would have decreased the relative blood flow to these regions and therefore, the [^18^F]NaF radiotracer uptake.

Overall, ULD [^18^F]NaF dPET imaging demonstrated the feasibility of marked radiotracer dose reduction without impairing diagnostic image quality. Additionally, the dPET image data sets were quantitatively assessed and ULD [^18^F]NaF dPET did not significantly underrepresent SUV_mean_ and SUV_max_ values when compared with LD and SD. As expected in a healthy canine, the average skeletal osteoblastic activity (i.e., SUV_mean_) showed no statistically significant differences among the 3 doses (Table 3). This further suggests that ULD [^18^F]NaF dPET is feasible. In addition, quantitative assessment in terms of SUV_max_ showed no statistically significant differences among SD, LD and ULD doses except in the first lumbar vertebra (Table 4). The first lumbar vertebra demonstrated SUV_max_ values significantly lower on SD when compared with LD or ULD but no significant differences in SUV_mean_ value were noted for these 3 doses. This may be due to an increase heterogeneity of [^18^F]NaF uptake in the lumbar vertebrae among subjects which the SUV_max_ will highlight and the SUV_mean_ will not. The caudal vertebrae (tail), which contains several small bones more distally located, showed no differences among doses in our qualitative and quantitative assessments. This further supports that ULD [^18^F]NaF dPET is sufficient for assessing normal osteoblastic activity even in small distal bones which are biomechanically active.

This study showed that using a clinical dPET system in a large animal model might provide guidance to perform translational studies that currently are only feasible in small laboratory animals using specialized micro PET/CT systems. A limitation of the study was that not all dogs received the three doses (ULD, LD, and SD); however, eight of the nine dogs received the ULD in addition to either the SD or LD. The use of a translational large animal model is expensive, requires extensive preparation and coordination, and presents more challenges when compared to smaller laboratory animals. Future studies will be needed to further assess this ULD [^18^F]NaF dPET/CT imaging approach for oncologic and non-oncologic osteoblastic diseases in preclinical large animal models.

Ultra-low dose [^18^F]NaF dPET/CT demonstrated a comparable diagnostic image quality and quantitative accuracy when compared with SD. This ULD dPET approach is consistent with the goals of ALARA in terms of minimizing radiation exposure not only to research subjects but also PET technologists, veterinary personnel, and caretakers.

## Data Availability Statement

The original contributions presented in the study are included in the article/Supplementary Material, further inquiries can be directed to the corresponding author/s.

## Ethics Statement

The animal study was reviewed and approved by the Institutional Animal Care and Use Committee (IACUC) of The Ohio State University.

## Author Contributions

MM and MK: study design and take responsibility for the integrity of the data analysis. MM, RM, and MK: study conduct. MM and RM: data collection. MM, RM, MA-R, SF, CW, and MK: data analysis. MM, MA-R, SF, RJ, and MK: data interpretation. MM: drafting manuscript. MM, RM, MA-R, SF, CW, RJ, and MK: revising manuscript content and approving final version of manuscript. All authors contributed to the article and approved the submitted version.

## Funding

This study was supported by Award Number Grant KL2TR002734 from the National Center for Advancing Translational Sciences, Ohio Third Frontier grants TECH 10-012, TECH 11-044, and TECH 13-060 and the Wright Center of Innovation in Biomedical Imaging Development Fund. REDCap electronic data capture tools hosted at The Ohio State University and supported by CTSA grant UL1TR002733. Funding bodies had no role in the design, data analysis, or writing of the manuscript.

## Conflict of Interest

The authors declare that the research was conducted in the absence of any commercial or financial relationships that could be construed as a potential conflict of interest.

## Publisher's Note

All claims expressed in this article are solely those of the authors and do not necessarily represent those of their affiliated organizations, or those of the publisher, the editors and the reviewers. Any product that may be evaluated in this article, or claim that may be made by its manufacturer, is not guaranteed or endorsed by the publisher.
