# Supplementary material for: [18F] Sodium Fluoride Dose Reduction Enabled by Digital Photon Counting PET/CT for Evaluation of Osteoblastic Activity
Source: Front Med (Lausanne). 2022 Jan 12;8:725118. doi: 10.3389/fmed.2021.725118 (PMC8789749; doi:10.3389/fmed.2021.725118)
Supplement: Supplementary file 1 [file Presentation_1.PPTX]

## Slide 1
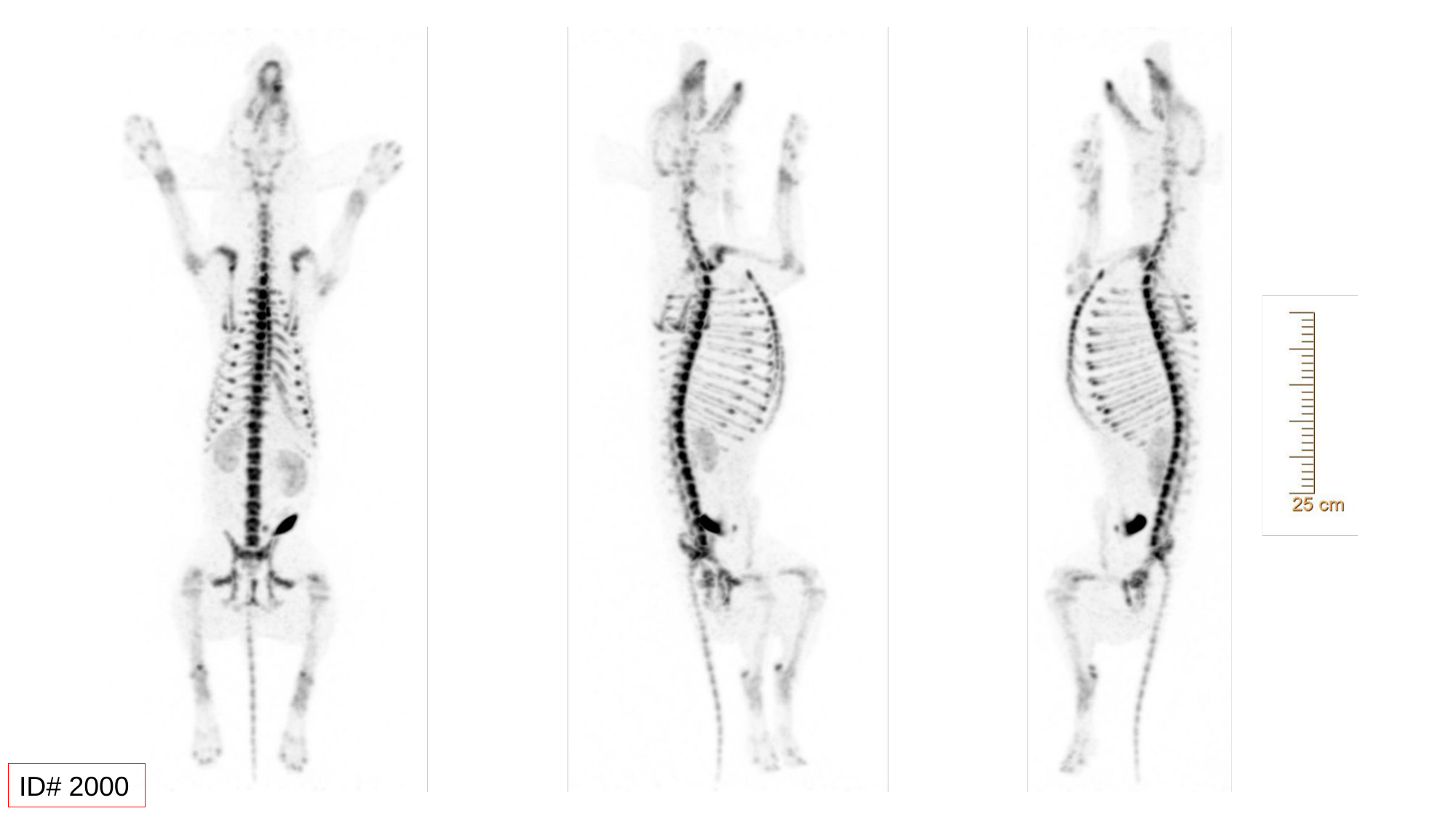

ID# 2000

## Slide 2
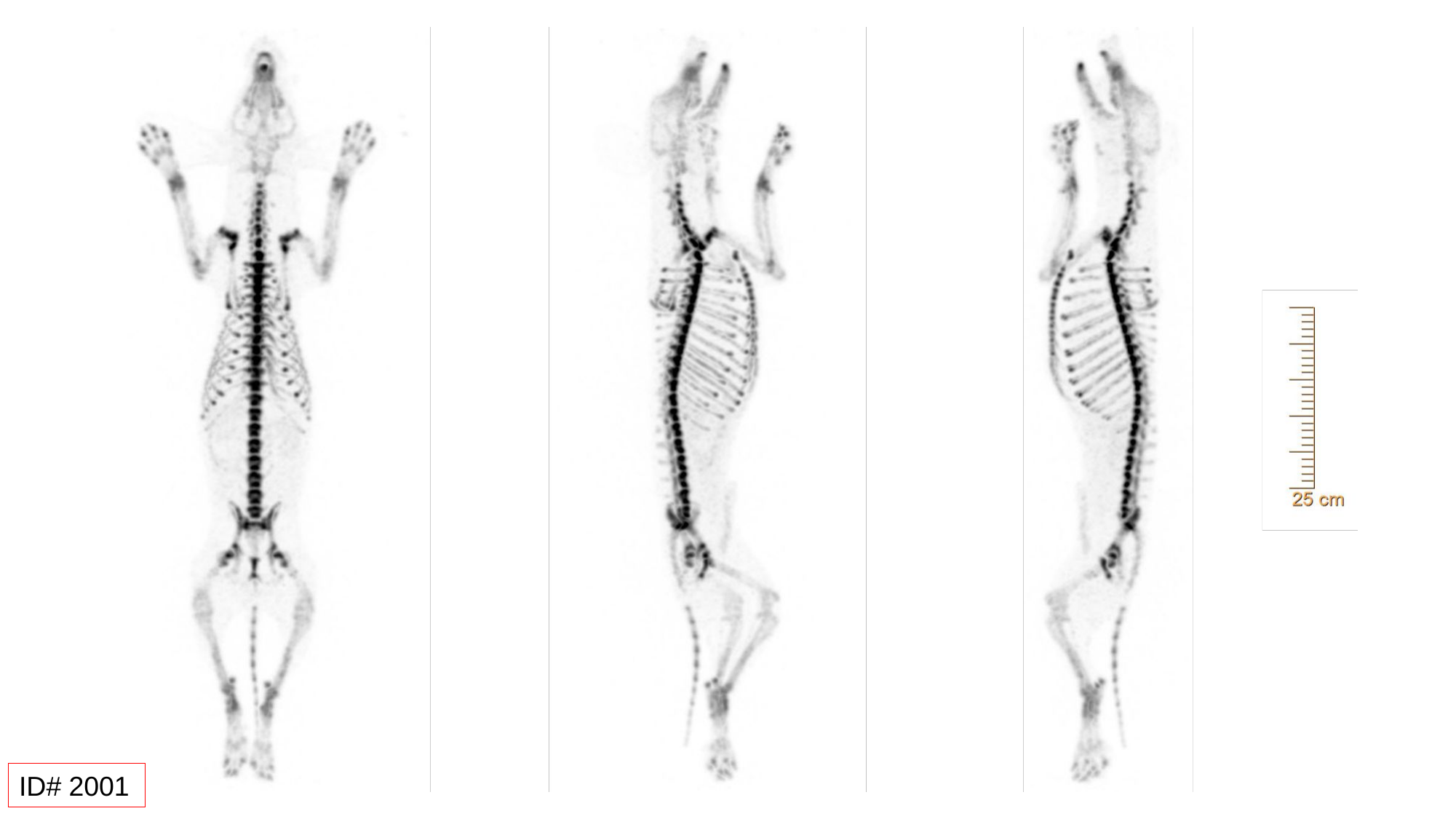

ID# 2001

## Slide 3
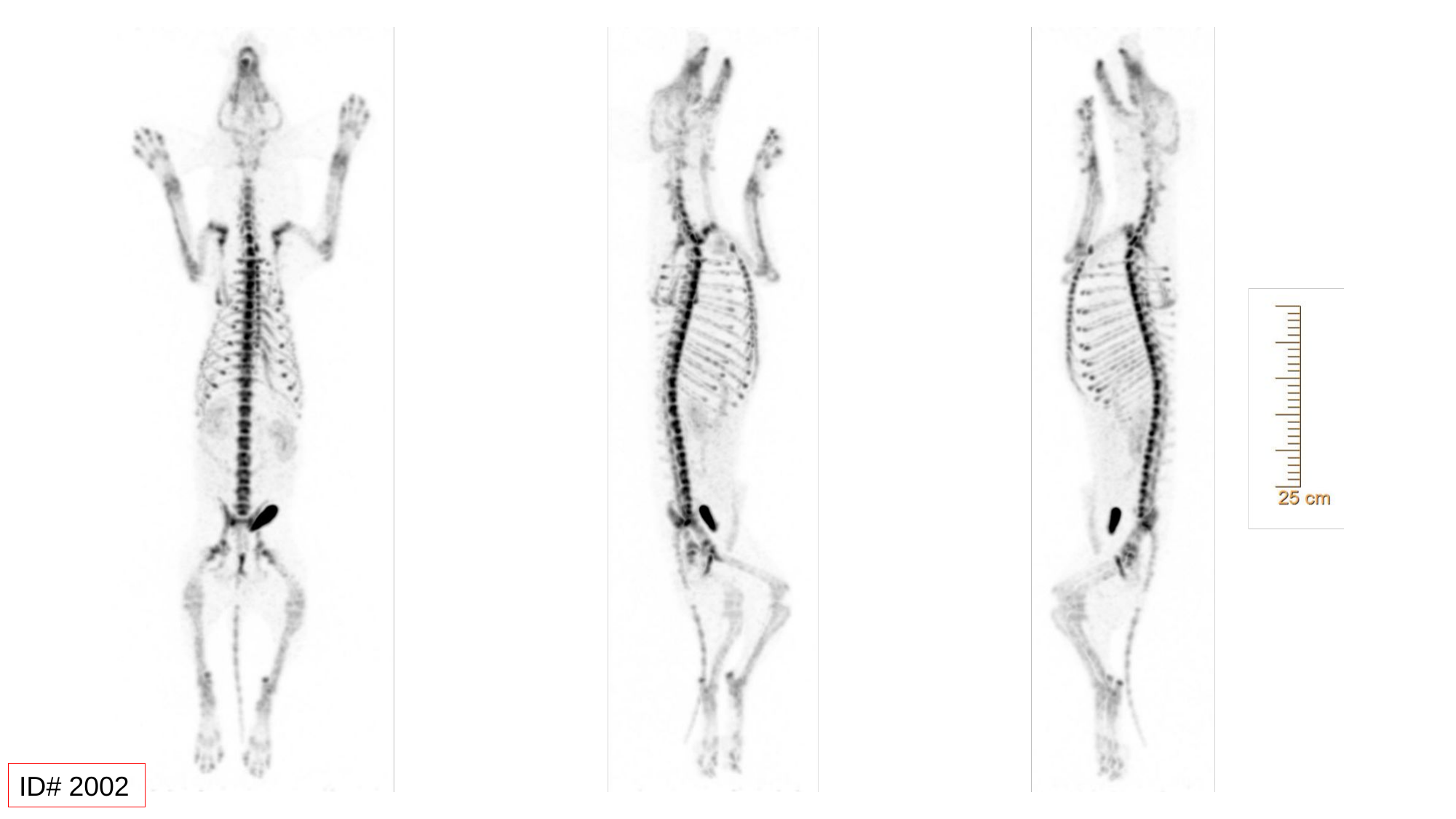

ID# 2002

## Slide 4
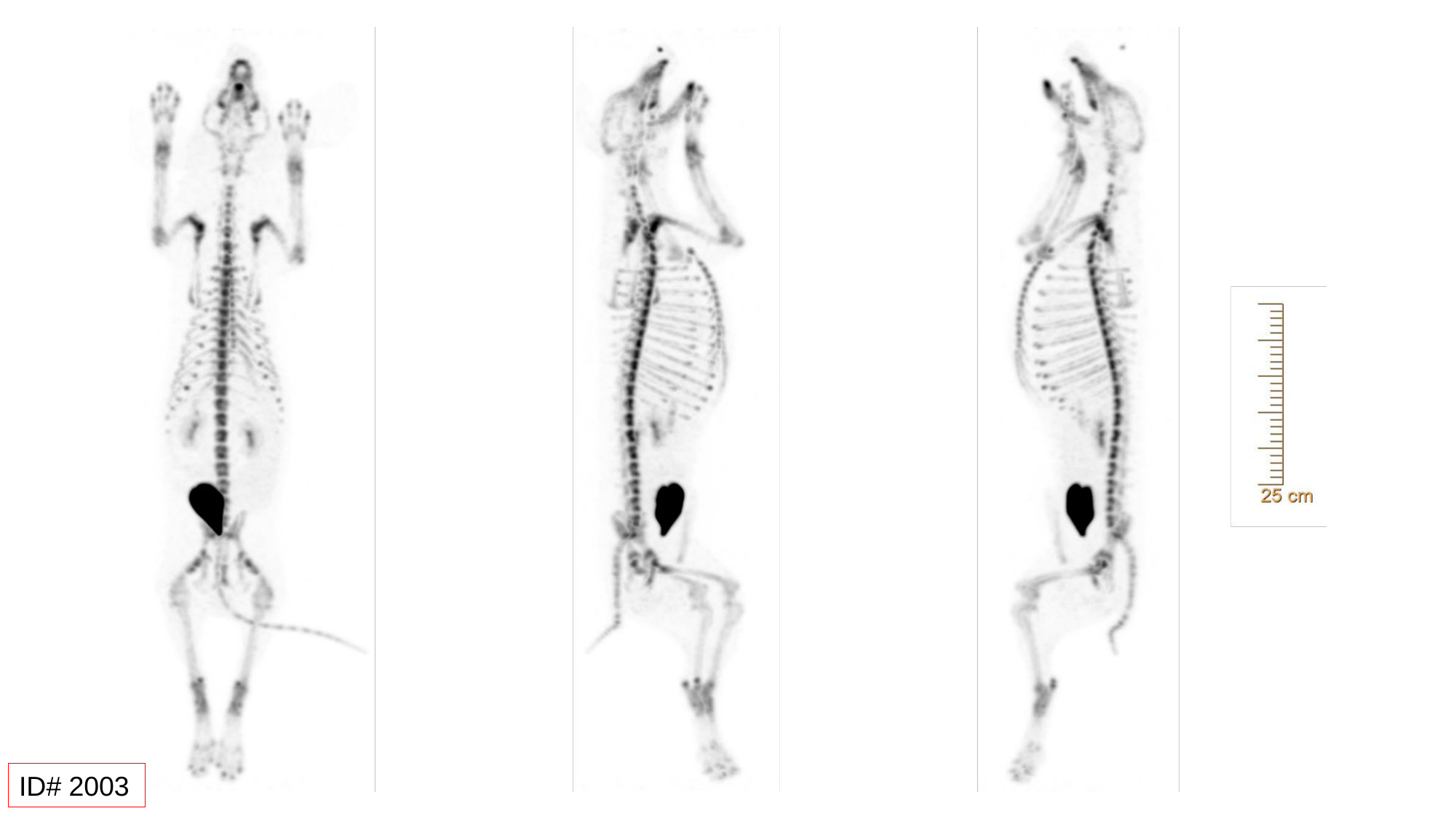

ID# 2003

## Slide 5
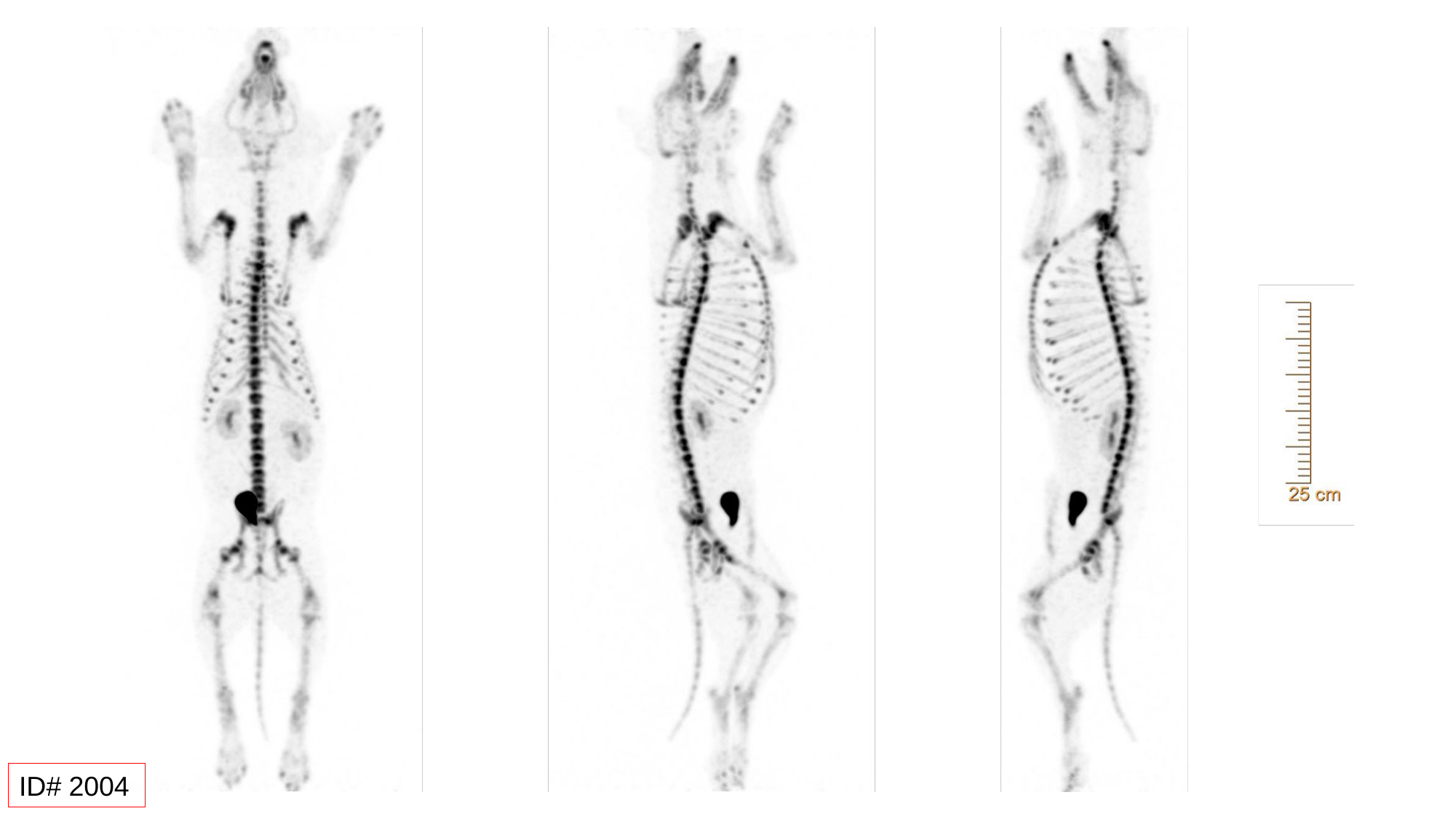

ID# 2004

## Slide 6
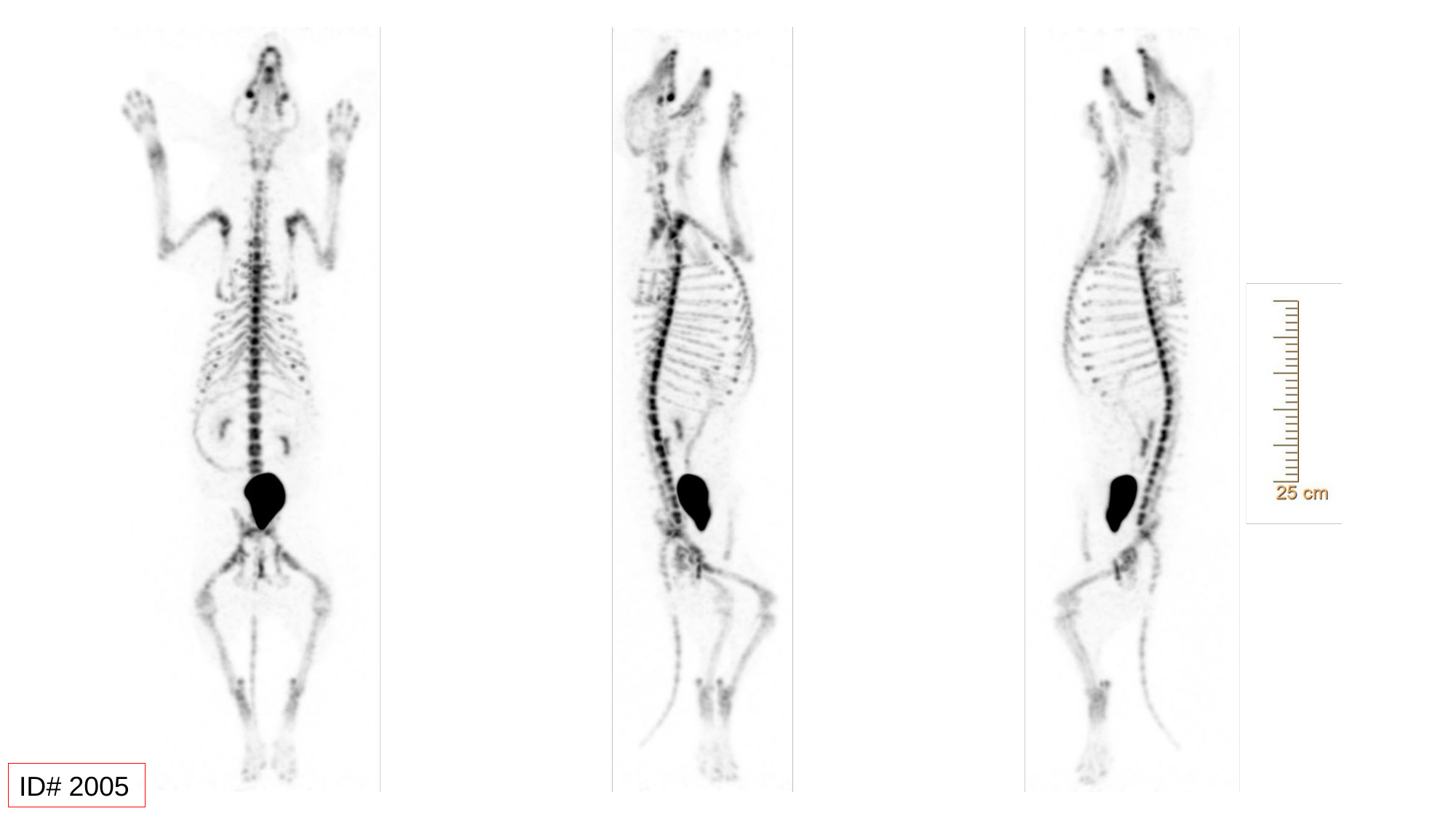

ID# 2005

## Slide 7
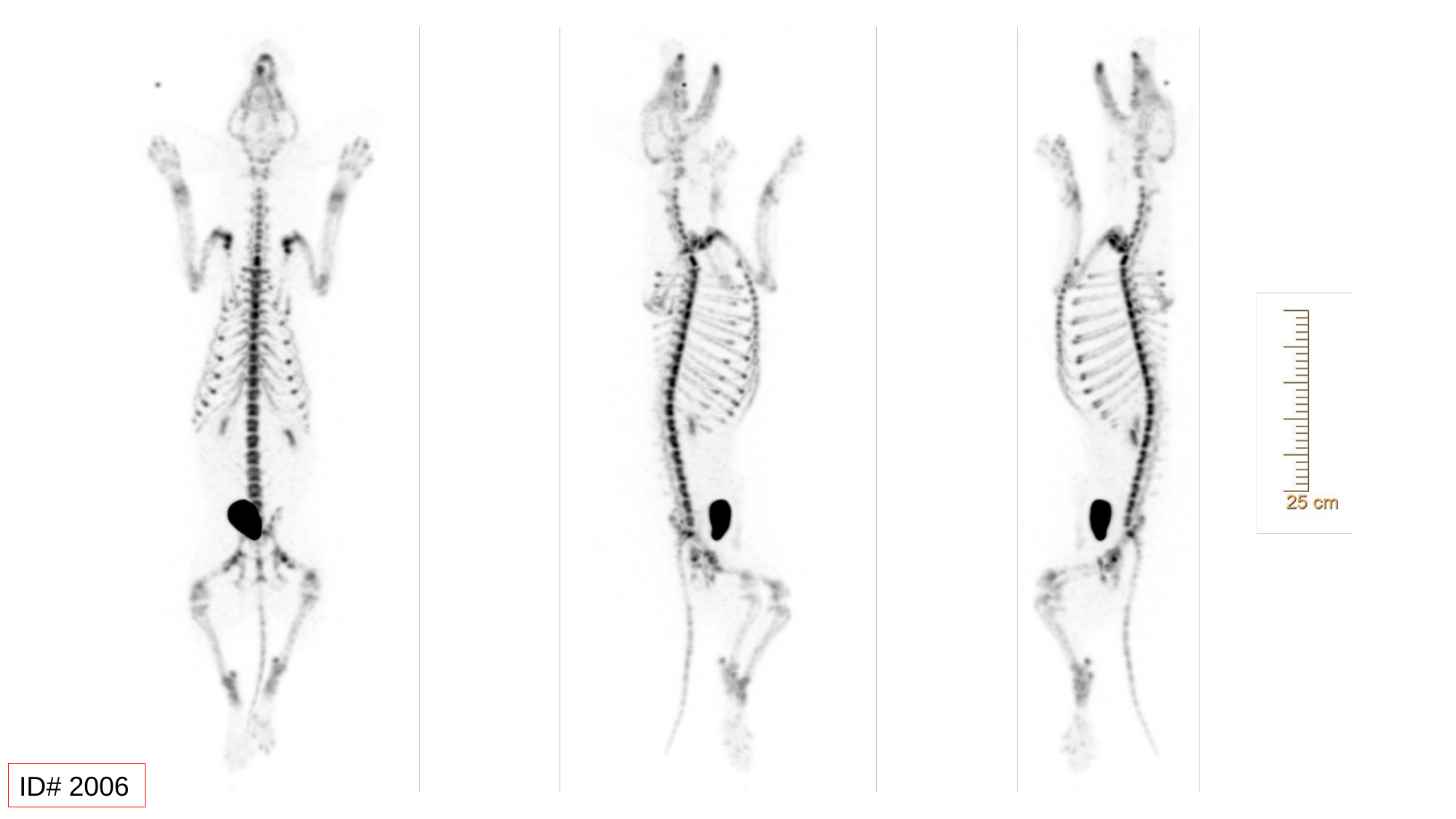

ID# 2006

## Slide 8
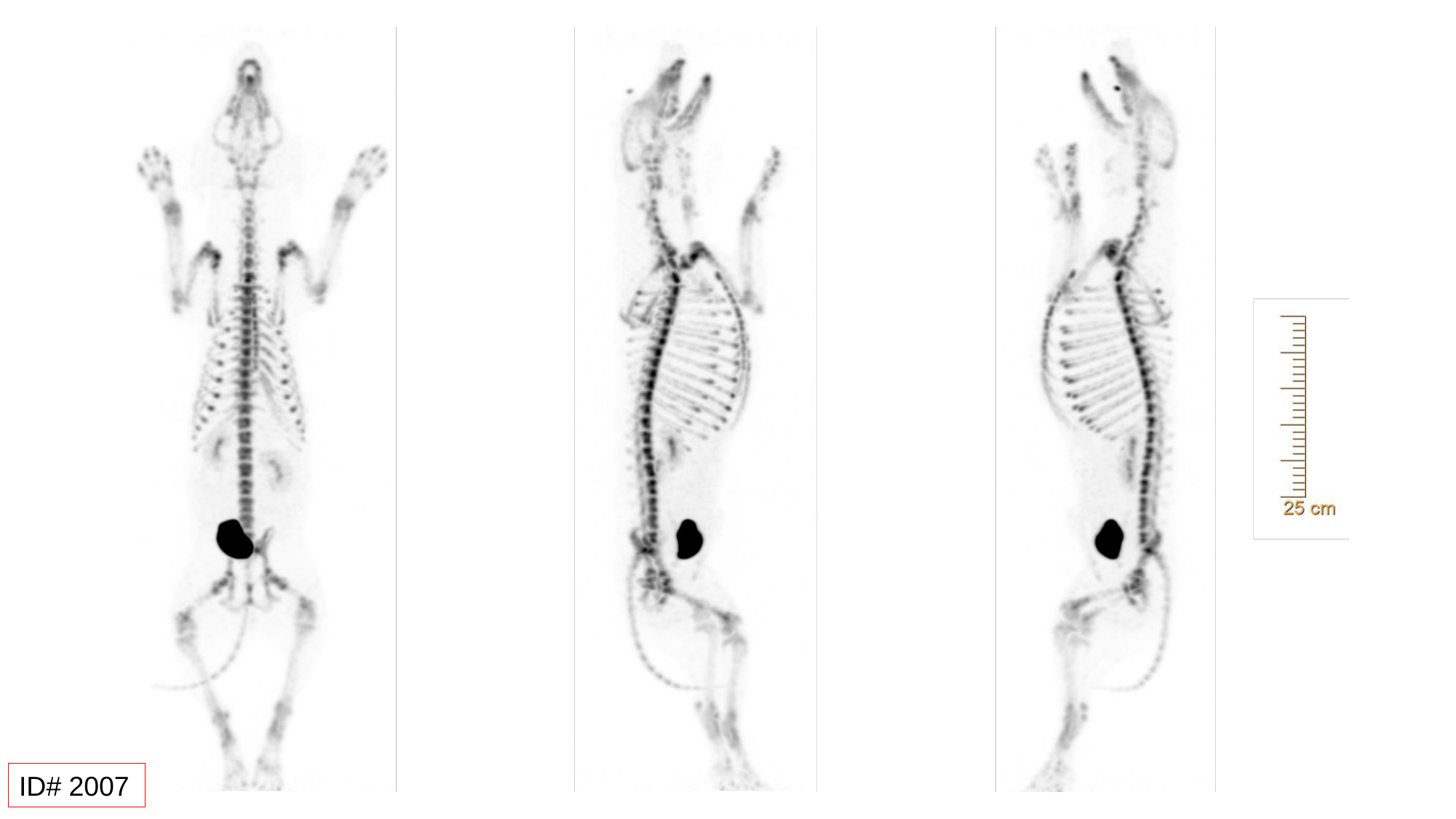

ID# 2007

## Slide 9
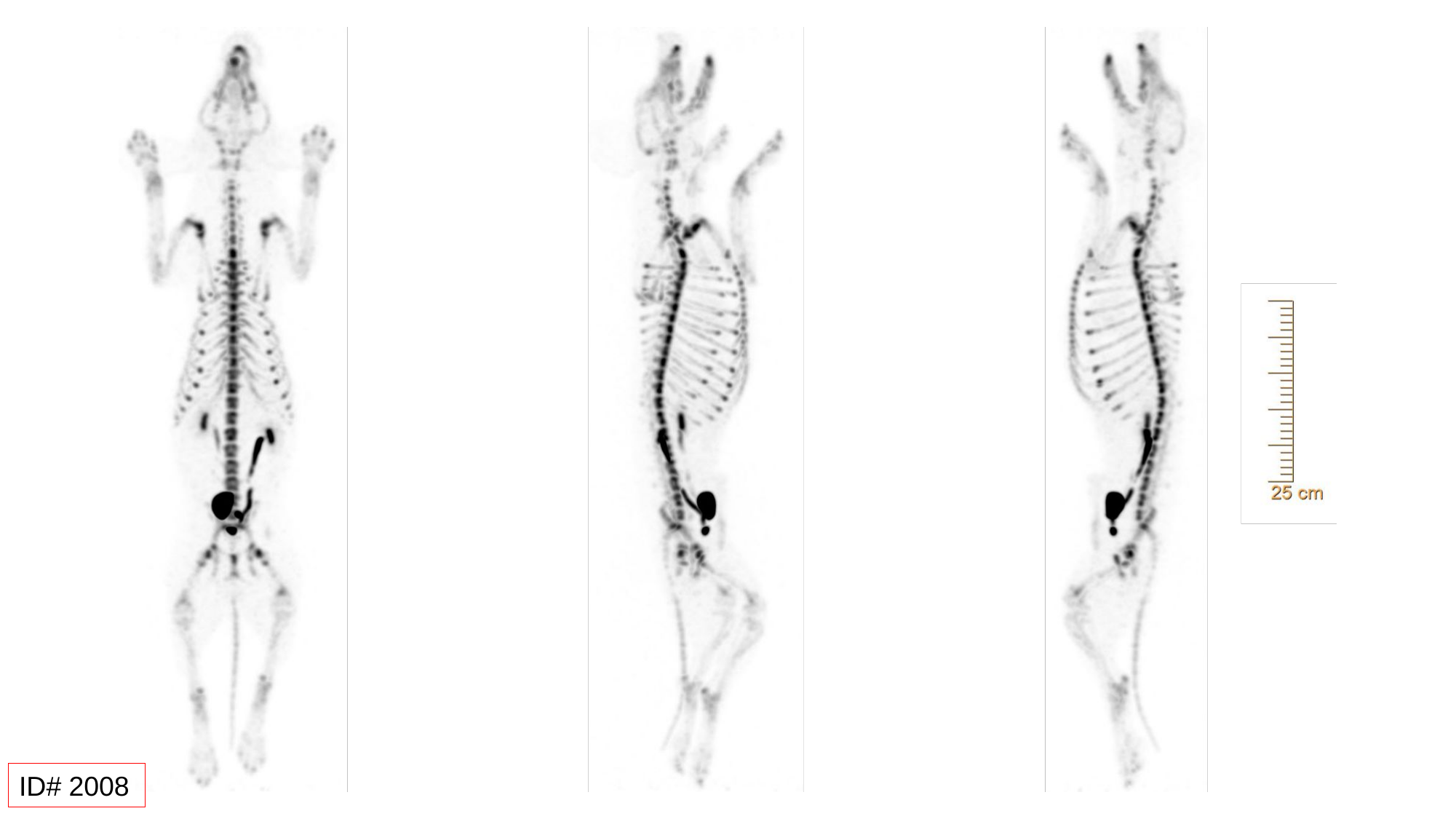

ID# 2008

## Slide 10
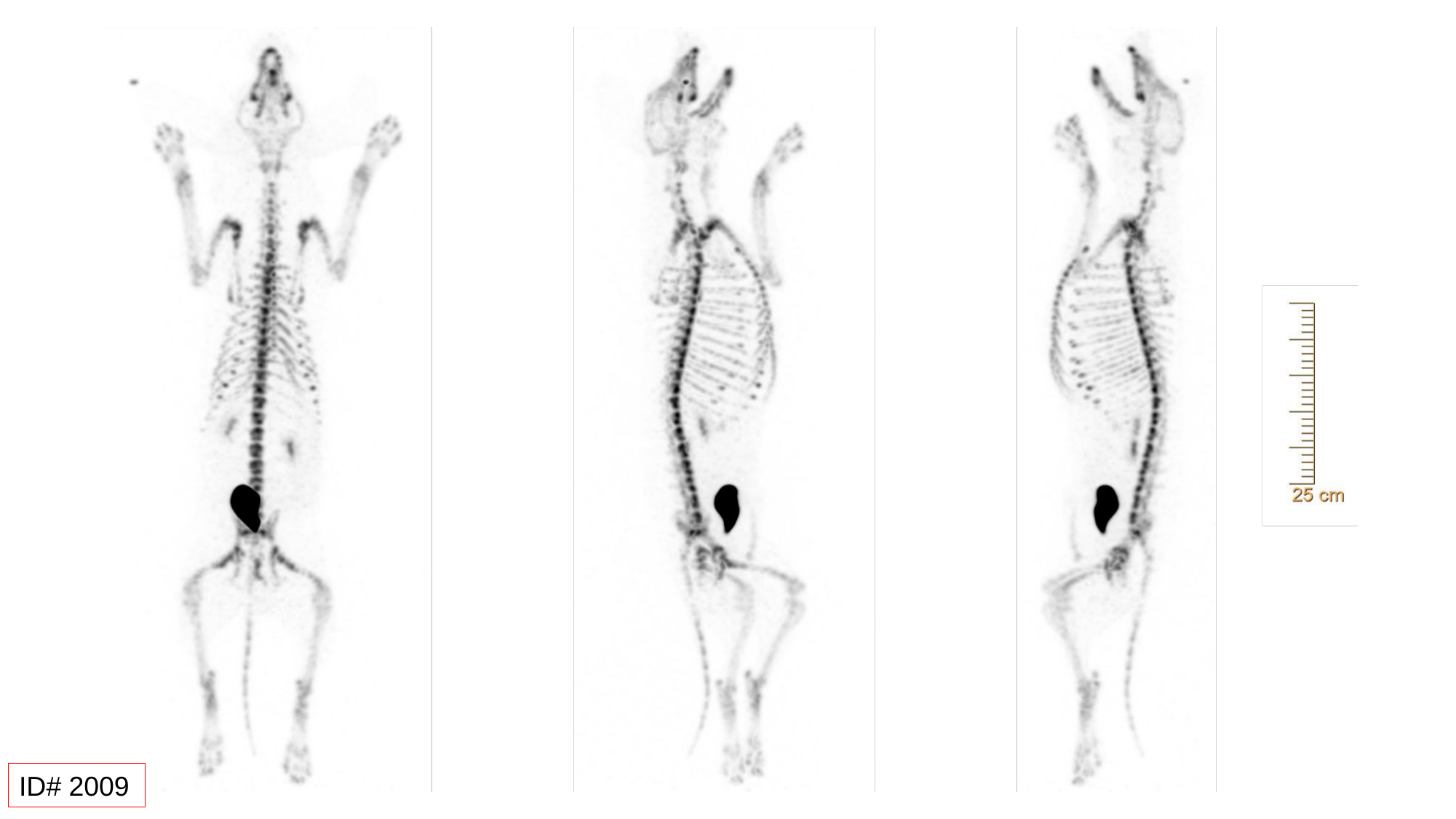

ID# 2009

## Slide 11
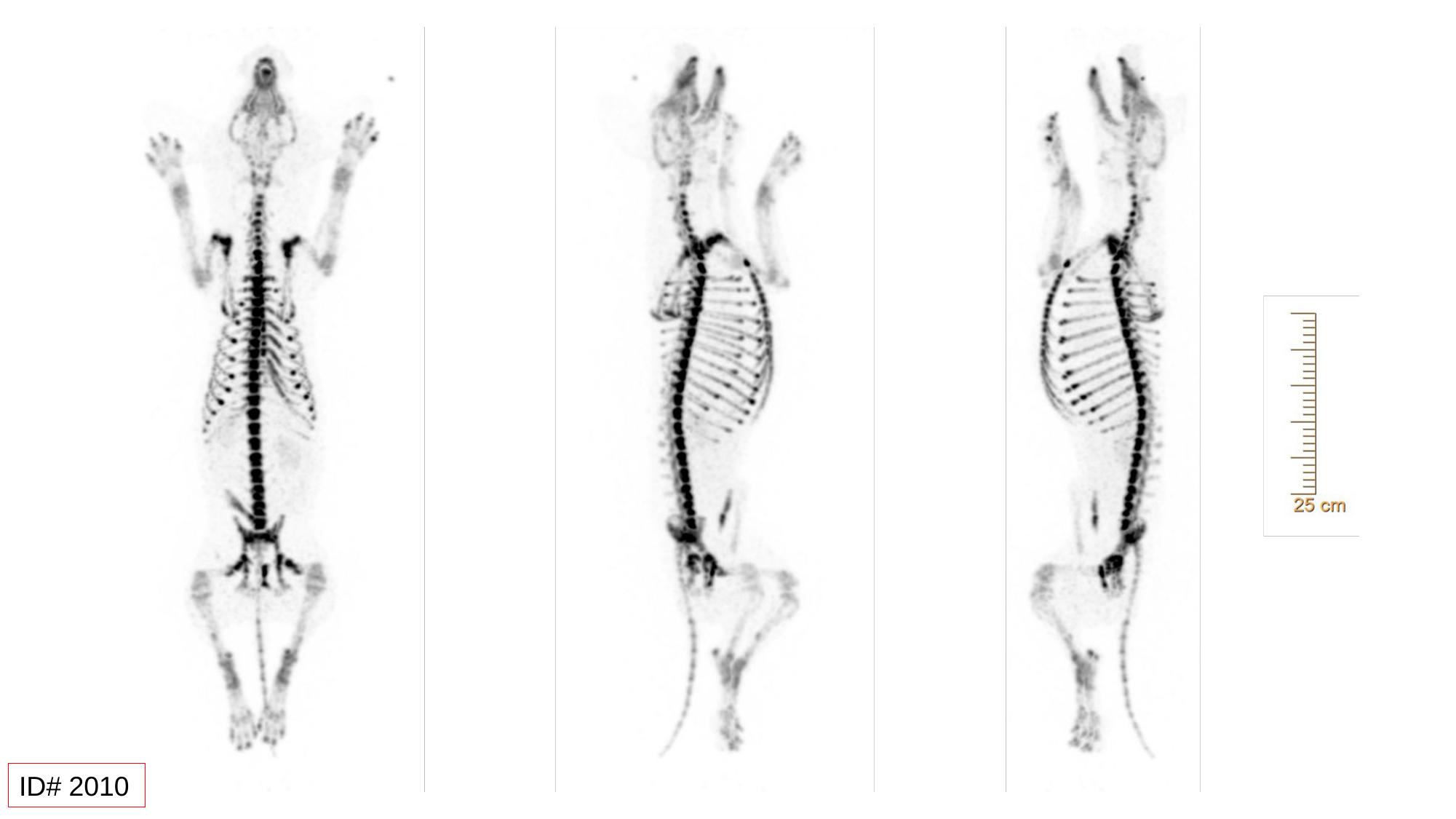

ID# 2010

## Slide 12
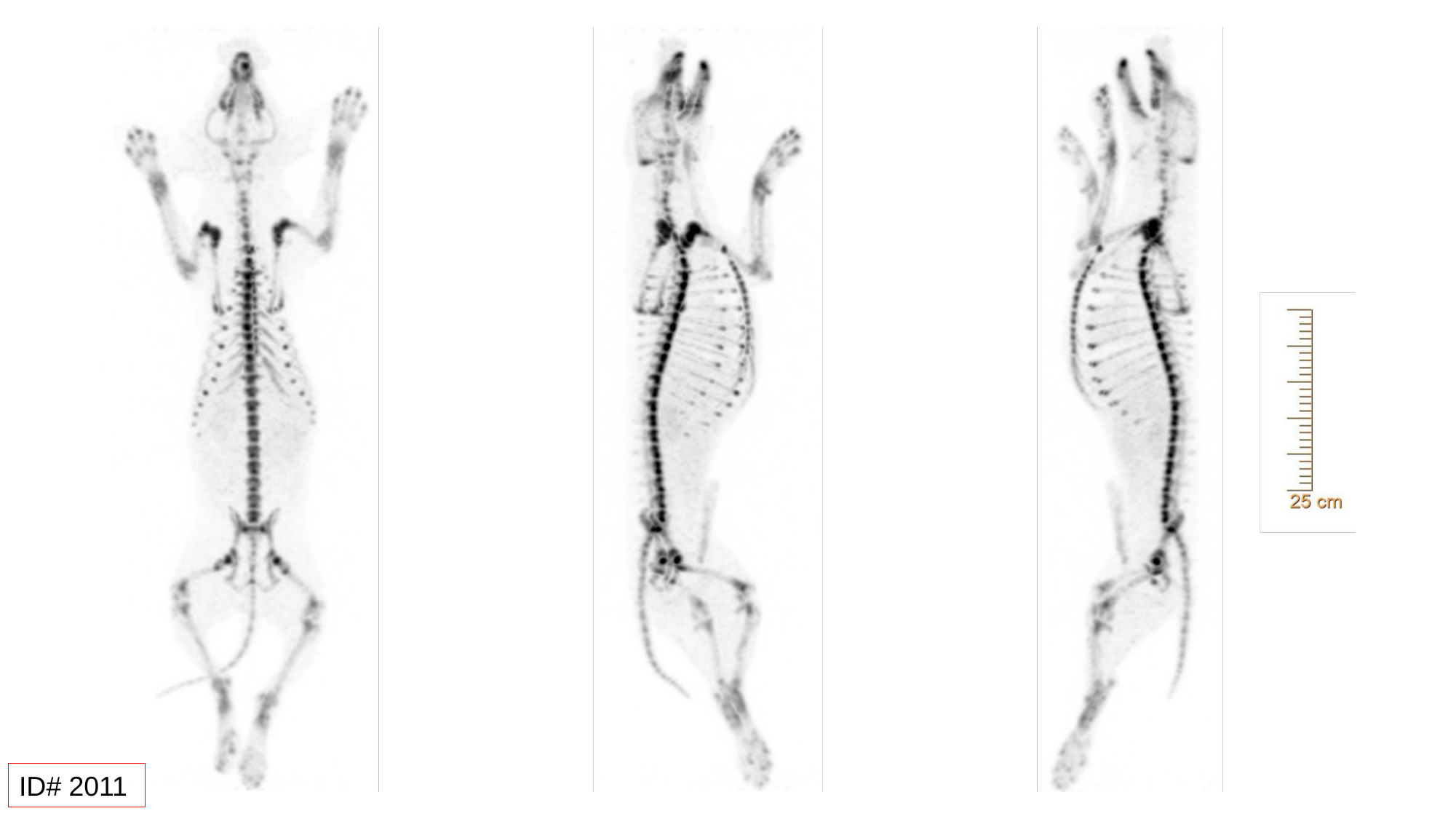

ID# 2011

## Slide 13
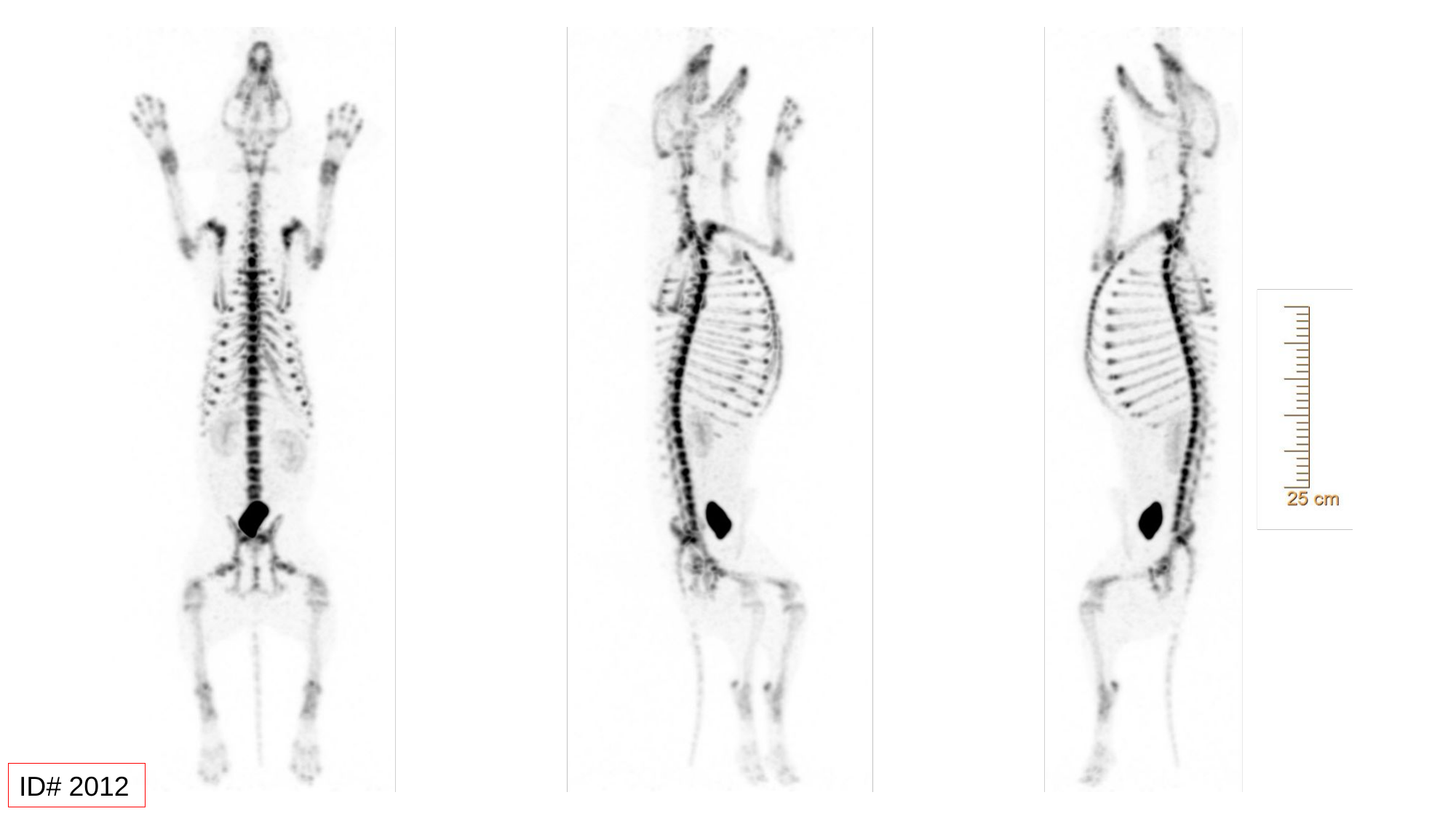

ID# 2012

## Slide 14
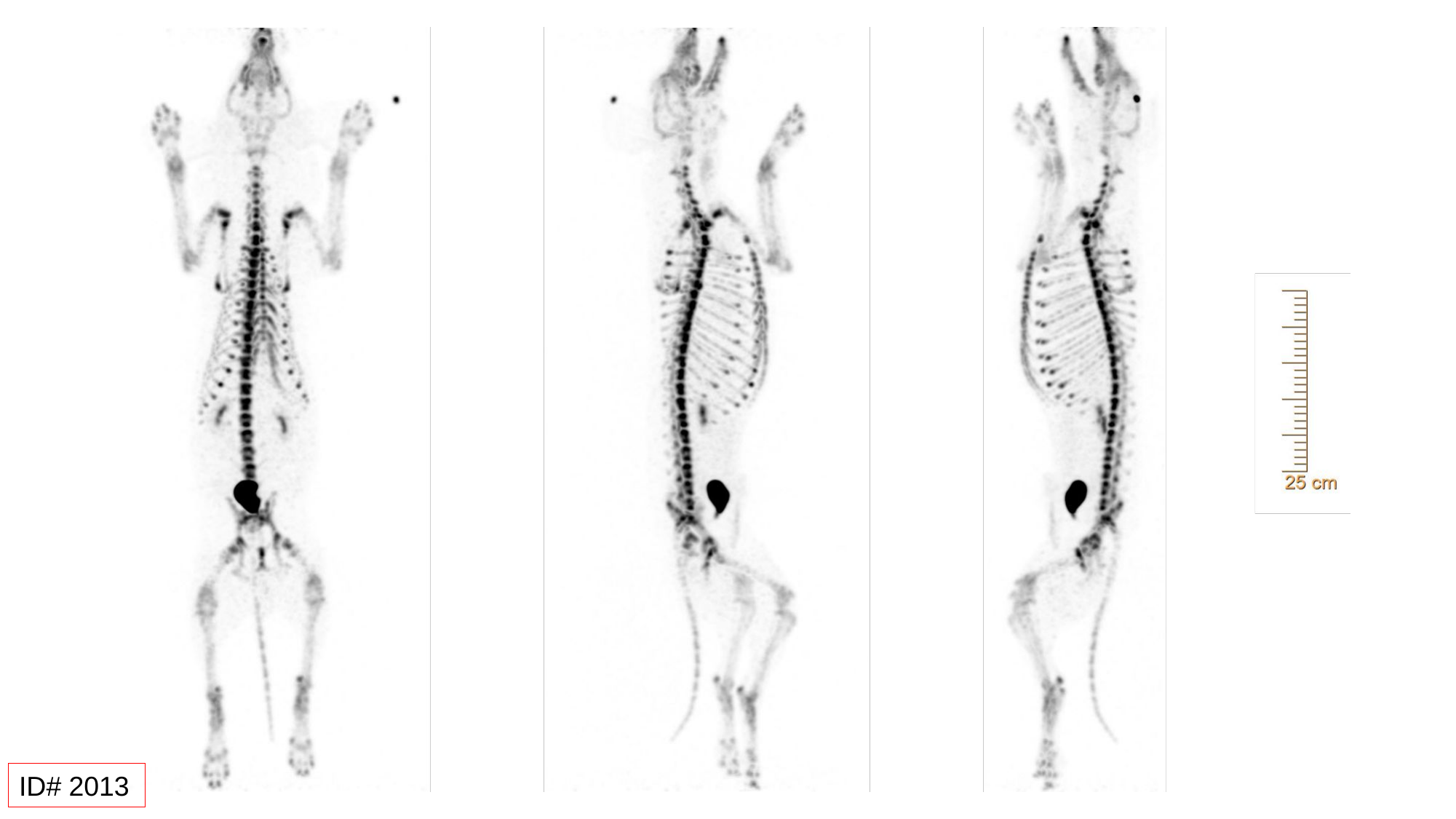

ID# 2013

## Slide 15
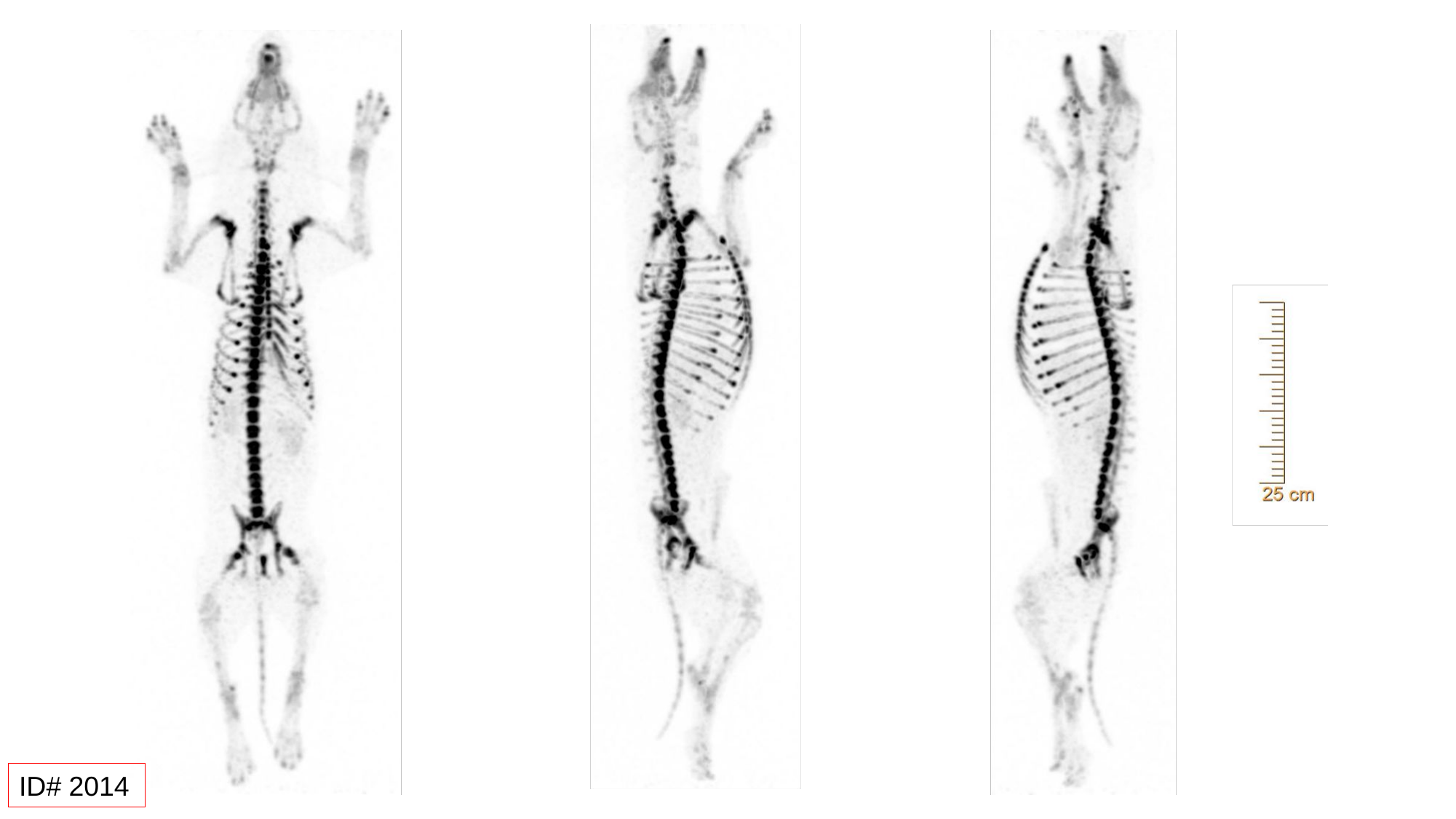

ID# 2014

## Slide 16
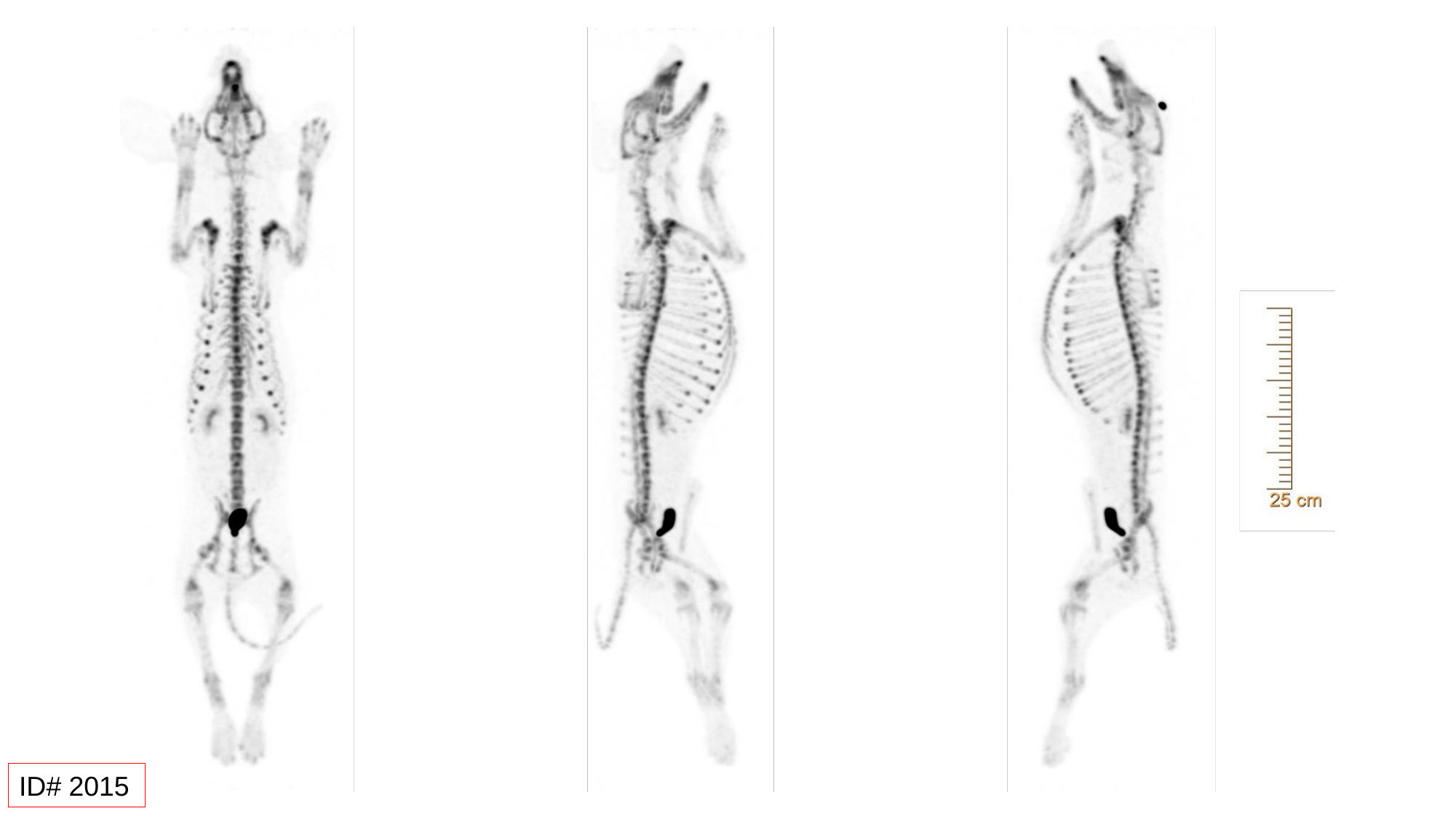

ID# 2015

## Slide 17
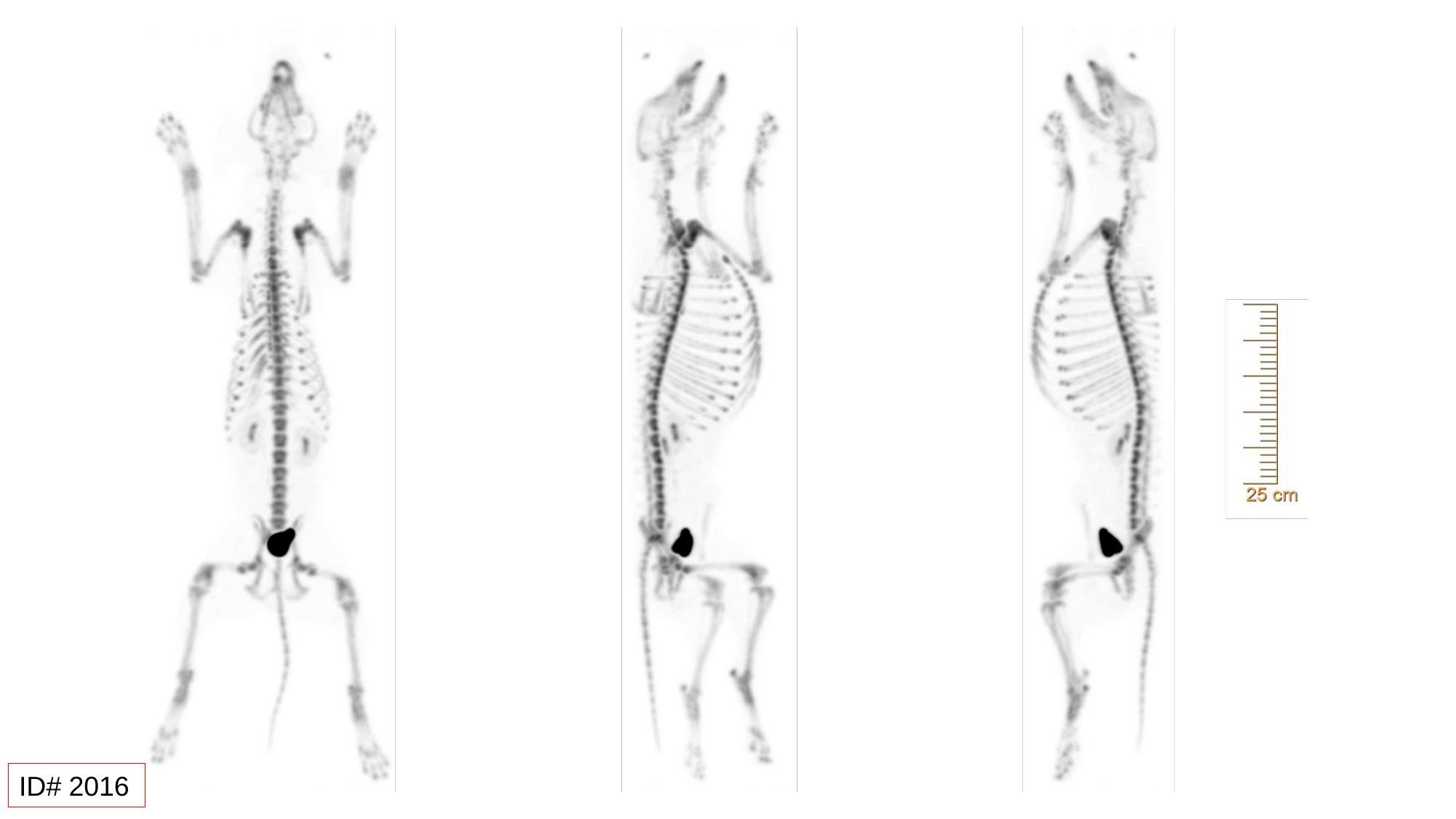

ID# 2016

## Slide 18
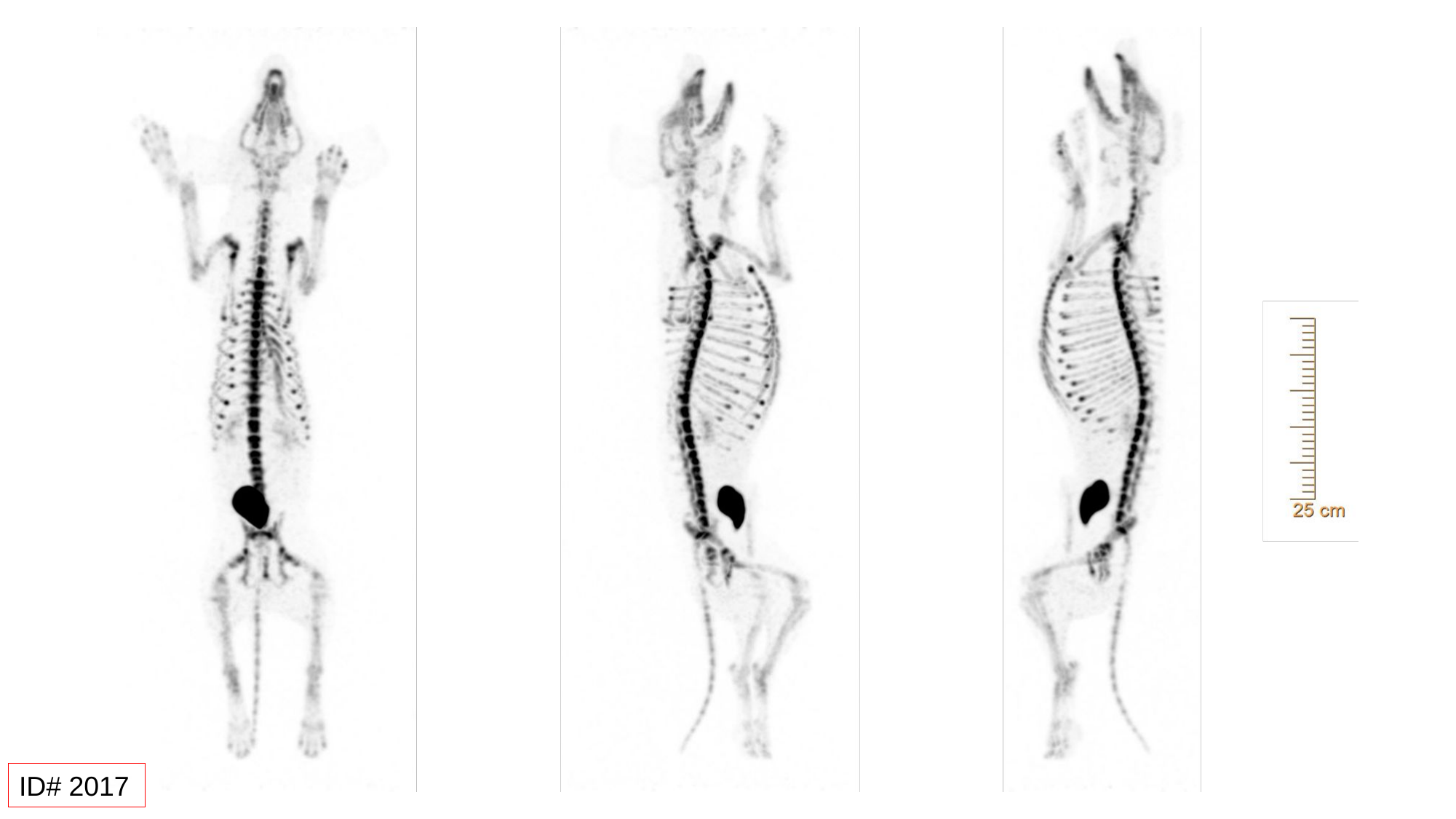

ID# 2017

## Slide 19
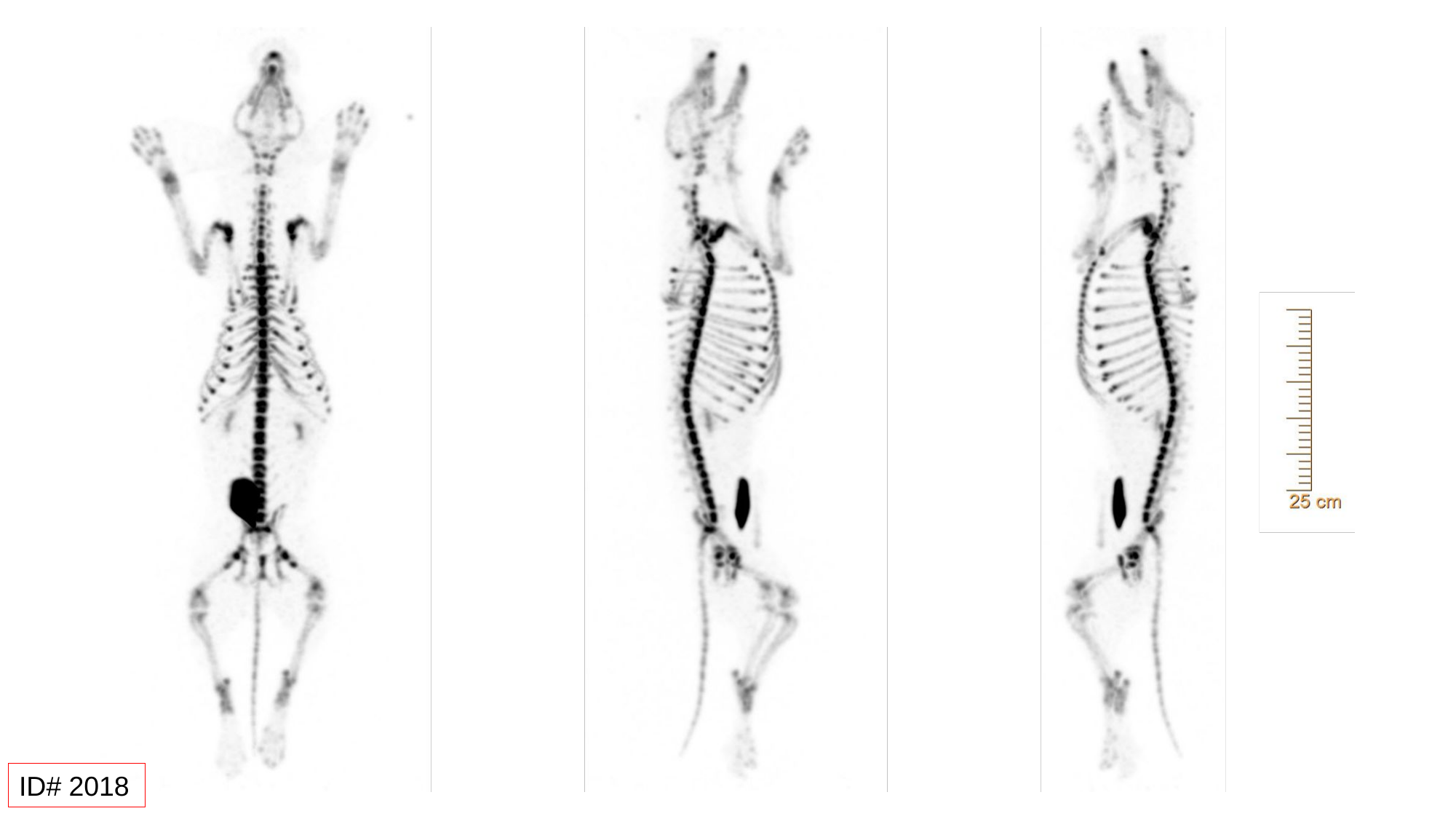

ID# 2018
