# Supplementary material for: [18F] Sodium Fluoride Dose Reduction Enabled by Digital Photon Counting PET/CT for Evaluation of Osteoblastic Activity
Source: Front Med (Lausanne). 2022 Jan 12;8:725118. doi: 10.3389/fmed.2021.725118 (PMC8789749; doi:10.3389/fmed.2021.725118)
Supplement: Supplementary file 2 [file Table_1.docx]

| **Dog** | **^18^F-NaF Dose (MBq)** | **RED Cap #** |
| --- | --- | --- |
| 113 | 1.9 | 2008 |
| 112 | 1.9 | 2014 |
| 111 | 1.9 | 2002 |
| 118_2 | 1.9 | 2012 |
| 118_1 | 1.9 | 2000 |
| 117 | 1.9 | 2017 |
| 116 | 1.9 | 2004 |
| 115 | 1.9 | 2013 |
| 114 | 1.9 | 2005 |
| 114 | 3.7 | 2009 |
| 113 | 37 | 2006 |
| 111 | 37 | 2001 |
| 112 | 18.5 | 2010 |
| 107 | 111 | 2016 |
| 114 | 111 | 2003 |
| 117 | 111 | 2015 |
| 116 | 111 | 2011 |
| 118 | 111 | 2007 |
| 113 | 3.7 | 2018 |

**Table 1: ^18^F-NaF Doses (MBq) and RED Cap survey number.**
